# Reducing Retail Merchandising of Discretionary Food and Beverages in Remote Indigenous Community Stores: Protocol for a Randomized Controlled Trial

**DOI:** 10.2196/12646

**Published:** 2019-03-29

**Authors:** Julie Brimblecombe, Megan Ferguson, Emma McMahon, Anna Peeters, Edward Miles, Thomas Wycherley, Leia M Minaker, Khia De Silva, Luke Greenacre, Catherine Mah

**Affiliations:** 1 Department of Nutrition, Dietetics and Food Monash University Notting Hill Australia; 2 Menzies School of Health Research Darwin Australia; 3 School of Public Health The University of Queensland Herston Australia; 4 Deakin University Geelong Australia; 5 Alliance for Research in Exercise, Nutrition and Activity University of South Australia Adelaide Australia; 6 School of Planning, Faculty of Environment University of Waterloo Waterloo, ON Canada; 7 Arnhem Land Progress Aboriginal Corporation Darwin Australia; 8 Department of Marketing Monash University Caulfield Australia; 9 Dalhousie University Halifax, NS Canada; 10 University of Toronto Toronto, ON Canada

**Keywords:** randomized controlled trial, indigenous population, food supply, diet

## Abstract

**Background:**

Discretionary food and beverages (products high in saturated fat, added sugars, and salt) are detrimental to a healthy diet. Nevertheless, they provide 42% of total energy and account for 53% of food and beverage expenditure for remote living Aboriginal and Torres Strait Islander Australians, contributing to the excessive burden of chronic diseases experienced by this population group.

**Objective:**

The aim of this study is to test an intervention to reduce sales of discretionary products, in collaboration with the Arnhem Land Progress Aboriginal Corporation (ALPA), which operates 25 stores in very remote Australia, by reducing their merchandising and substituting with core products in remote Australian communities.

**Methods:**

We will use a community-level randomized controlled pragmatic trial design. Stores randomized to the intervention group will be supported by ALPA to reduce merchandising of 4 food categories (sugar, sugar-sweetened beverages, sweet biscuits, and confectionery) that together provide 64% of energy from discretionary foods and 87% of total free sugars in very remote community stores. The remaining stores (50% of total) will serve as controls and conduct business as usual. Electronic store sales data will be collected at baseline, 12-weeks intervention, and 24-weeks postintervention to objectively assess the primary outcome of percent change in purchases of free sugars (g/megajoule) and secondary business- and diet-related outcomes. Critical to ensuring translation to improved store policies and healthier diets in remote Indigenous Australia, we will conduct (1) an in-depth implementation evaluation to assess fidelity, (2) a customer intercept survey to investigate the relationship between customer characteristics and discretionary food purchasing, and (3) a qualitative study to identify policy supports for scale-up of health-enabling policy action in stores.

**Results:**

As of August 2018, 20 stores consented to participate and were randomized to receive the intervention or continue usual business. The 12-week strategy ended in December 2018. The 24-week postintervention follow-up will occur in May 2019. Trial results are expected for 2019.

**Conclusions:**

Novel pragmatic research approaches are needed to inform policy for healthy retail food environments. This research will greatly advance our understanding of how the retail food environment can be used to improve population-level diet in the remote Australian Aboriginal and Torres Strait Islander context and retail settings globally.

**Trial Registration:**

Australian New Zealand Clinical Trials Registry ACTRN12618001588280; http://www.anzctr.org.au/Trial/Registration/TrialReview.aspx?id=375933 (Archived by WebCite at http://www.webcitation.org/76dbQEmwN)

**International Registered Report Identifier (IRRID):**

DERR1-10.2196/12646

## Introduction

### Background

Aboriginal and Torres Strait Islander people residing in remote Australian communities bear a disproportionate burden of preventable chronic diseases [1]. Poor diet quality, including excessive intake of discretionary food and drinks, is a major contributor to preventable chronic diseases for all Australians [2]. Discretionary products are those that are not necessary for a healthy diet and are high in saturated fat, added sugars, and salt [3]. They are detrimental to a healthy diet as they displace more nutritious core (nondiscretionary) foods. Nevertheless, for the Aboriginal and Torres Strait Islander population living in remote Australian communities, discretionary products provide 42% of total energy [4] and account for 53% of food and beverage expenditure [5]. Reducing discretionary product intake is imperative to improving health in this population group.

The majority of food consumed in remote communities is purchased at the local community store [6]. Community stores, in most instances, belong to the community, which gives members the power to initiate and sustain community-level change. Optimizing the store environment as a health-enabling setting, in partnership with the community, represents a key strategy and opportunity to improve dietary quality and reduce preventable chronic disease burden.

We recently led the “stores healthy options project in remote indigenous communities (SHOP@RIC)” study, a large trial with 20 remote community stores to assess the impact of a price discount on purchasing [5]. It responded to concerns expressed by community Aboriginal leaders that high food prices of core items were driving a diet dominated by unhealthy foods and beverages as these provided cheap calories, tasted good, and were convenient and easy to store [7,8]. We found that a price discount on fruit and vegetables increased their purchase. However, any potential positive health gains, may have been negated because of the concomitant increase in the purchases of other foods including both core and discretionary products [9]. This may be explained by shoppers redirecting their produce savings toward those readily available and well-merchandised items. This evidence supports findings from a systematic review of grocery store interventions and the food price modeling literature, which conclude that discounts on healthy core food groups need to be accompanied by parallel pricing or strategies to reduce purchasing of discretionary products and encourage healthier food purchasing [10-12]. At the end of the SHOP@RIC trial, in a knowledge exchange meeting, retail leaders working with remote community stores and community store directors recommended that we collaborate to test how merchandising strategies could optimize the store environment to encourage healthier food purchasing.

Merchandising is the “activity of promoting the sale of goods, especially by their presentation in retail outlets,” and it includes activities such as display techniques, free samples, pricing, shelf talkers, and other point-of-sale methods [13]. Merchandising utilizes the 4 elements of marketing management (ie, product, price, promotion, and place) to competitively position a product in the marketplace [14]. In this planned trial, we will use a pragmatic community-level randomized controlled trial to assess the impact on consumer purchasing and retail performance of a population-level intervention that targets discretionary products through reducing their merchandising in remote Aboriginal and Torres Strait Islander Australian community stores. We will target 4 product types (sugar per se, sugar-sweetened beverages, sweet biscuits, and confectionery) that were together shown to contribute 64% of the energy from discretionary product purchases and 87% of free sugars (free sugars include all sugars added to products plus sugars naturally present in honey, syrups, and fruit juices), using purchasing data from 20 remote community stores [5]. This is a novel approach; specifically targeting discretionary product purchases through modifying the store environment rather than solely focusing on increasing access to and promotion of core (healthier) food and beverages has rarely been a focus for public health interventions [15].

Our research will increase the understanding of merchandising as a factor influencing dietary behavior in consumer food environments [14,16], and one that, with appropriate support, is modifiable by retailers. We will conduct this research in collaboration with the Arnhem Land Progress Aboriginal Corporation (ALPA), one of the largest remote retail store associations and employers of Aboriginal and Torres Strait Islander people in Australia. This research will advance our knowledge on the implementation and optimization of pragmatic retail food environment interventions to enhance population-level diet. It will translate directly into practice and policy through a policy analysis and the involvement of key stakeholders (including remote community leaders and remote community store directors) through evidence synthesis and knowledge exchange on policy options.

### Objectives

Our study objectives are to:

1. assess the impact of reducing discretionary product merchandising on customer purchasing and retail business performance in remote Indigenous communities,
2. identify characteristics of customers associated with discretionary product purchasing, and
3. analyze and characterize the policy supports needed to scale-up nutrition evidence uptake in retail stores in remote Indigenous Australia.

This study tests the hypotheses that, over a 12-week intervention (and at 24-weeks postintervention), a strategy designed to reduce the merchandising of target discretionary products will reduce grams of free sugars per megajoule (MJ) energy (ie, sugars added to products plus sugars naturally present in honey, syrups, and fruit juices) in foods and drinks purchased through the community store (our primary outcome measure), and it will have positive impacts on secondary outcome measures relating to business performance and diet. This paper will describe the detailed protocol for study aims 1 and 2, and it excludes the policy analysis, aim 3.

## Methods

### Setting

Aboriginal and Torres Strait Islander people represent 3.3% of the Australian population [17]. A total of 19% of Aboriginal and Torres Strait Islander people live in remote or very remote areas of Australia in small towns commonly referred to as communities and/or homelands. These communities vary in size with most having fewer than 1000 people. Remote and very remote areas in Australia are defined by an objective measure of relative access to services on the basis of geographic distance to service centers [18,19]. According to the socioeconomic index for areas, these communities are also considered to be socioeconomically disadvantaged on the basis of aggregated social and economic information collected through the national Australian census [20]. In remote and very remote Aboriginal and Torres Strait Islander communities, the most amount of food is acquired from the local community retail store. There are over 170 community food retail stores throughout Australia [21]. These are small- to medium-sized retail businesses [22], in many cases, owned by the community, and these are major employers of local Aboriginal and Torres Strait Islander people [23]. In the northern territory (NT), the Australian Government Department of Prime Minister and Cabinet (PM&C) is responsible for community store licensing legislation under the Stronger Futures in the NT Act 2012 [24]. Licensees are required to stock a satisfactory range of healthy and good quality food, drink, and grocery items and demonstrate reasonable steps to promote food nutrition and health products. Other aspects of a store’s operations that may impact food security are also considered within the license.

ALPA is Australia’s largest Indigenous corporation [25]. ALPA was formed in 1972 and has grown to be a large-sized corporation employing 1100 people. ALPA owns 7 stores in 6 communities in North East Arnhem Land, NT, where the community members are ALPA shareholders. In addition, ALPA, manages 13 stores in the NT and Queensland through its Australian Retail Consultancy arm that was set up in 2002. In 2013, it acquired the Island and Cape chain of 6 stores in Cape York and the Torres Strait Islands in North Queensland. It also operates businesses that aim to create local employment. ALPA has long recognized the importance of promoting health and nutrition in the communities it serves, and its food and nutrition policy first implemented in the early 1980s aims to increase the availability and affordability of nutritious foods and the understanding of health, good food, and nutrition among its customers [26]. For example, for over 30 years, ALPA has subsidized fruit and vegetables, and it now provides discounted 600 ml bottled water at Aus $1. In 2017, ALPA developed a front-of-store and end-of-aisle strategy to be a part of its nutrition policy that reduces merchandising of discretionary products. ALPA expressed its desire for this strategy to be rigorously evaluated. The Healthy Stores 2020 intervention was co-designed with ALPA, and it incorporates elements of the ALPA front-of-store and end-of-aisle strategy. Like ALPA, local store board directors in many remote Indigenous communities are increasingly receptive to considering strategies, in some cases, actively modifying the store environment, to discourage discretionary product purchases and encourage healthy food and drink purchases [27,28].

### Design

**Figure 1:**
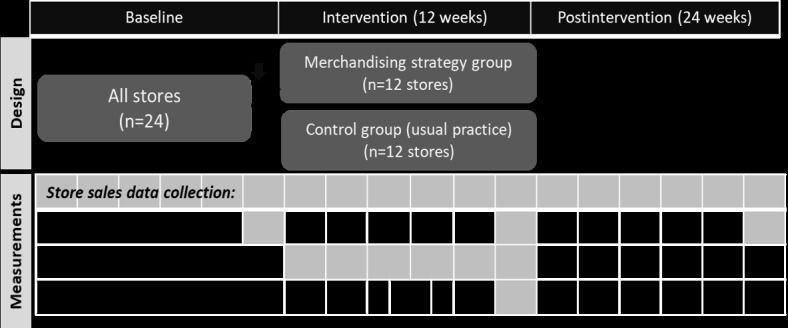
Study design schema.

We will use a community-level pragmatic randomized parallel group, 2-arm, superiority trial with a 1:1 allocation ratio design and a baseline, 12-week intervention period and 24-week postintervention period to assess the effect of the intervention on customer purchasing and store business performance measured objectively through store sales data (Figure 1). We will use a customer intercept survey to address our second aim of determining the characteristics of customers associated with discretionary product purchasing. An in-depth implementation evaluation will assess implementation fidelity (ie, intervention compliance) using a Merchandising Checklist administered fortnightly by phone with store managers and accompanying in-store photographs captured by store managers. A Store Environment Tool “Store Scout” (developed by Menzies School of Health Research) will be used to assess changes to the retail choice architecture and contextualize the results.

Our intervention has been co-designed with ALPA, and it will reduce the merchandising of high sugar discretionary products and subsequent desirability of these products, while allowing for substitute merchandising of core foods. Our overarching aim is to reduce the purchasing of targeted discretionary items. Due to the unknown impact of the intervention on business outcomes including level of resources needed for full implementation, ALPA considered a 12-week intervention period as acceptable. We previously reported short-term (ie, less than 6 months) food price interventions to be effective when applied in stores and/or supermarkets [12]. For this study, we considered a 12-week intervention to be of adequate duration to demonstrate the impact assuming immediate customer response and implementation compliance. We found in a previous trial that a 24-week postintervention period allowed an assessment of longer-term intervention impact on store practice without losing the engagement of key stakeholders.

### Theoretical Framework

The strategy is informed by a social ecological theory that poses that behavior is shaped by interaction between the individual and the environment [29]. Using this theory, we assert that specific factors in the retail food environment can incentivize and drive the excessive consumption of discretionary products. Therefore, to modify behavior, it is necessary to focus on modifying its determinants in the store food choice architecture rather than on individuals alone (eg, through customer education). We have drawn on consumer decision-making models to understand the use of merchandising practices by retailers [30-32]. These models generally consider food choice decisions as a 3-staged process of (1) product awareness, (2) interest or desire, and (3) the decision to buy or not. The merchandising techniques used by retailers aim to influence this process through increasing visibility (ie, awareness) and attraction (ie, interest or desire) of brands and products at point-of-sale to stimulate customers’ purchases, especially impulse purchases, which can make up 46% or more of total purchases [31-33]. An impulse purchase is one that occurs without a previously recognized need for the item [30], and the likelihood of making an impulse purchase is influenced by both product characteristics (eg, hedonicity and price) and retailer variables (eg, price, promotion, and place) [30]. Discretionary products are highly palatable, often available in a ready-to-eat form and have high hedonic appeal (ie, elicit prompt pleasurable emotions). As consumers tend to forego long-term negative results for immediate gratification, discretionary products are commonly impulse purchases [31] as the mix of desirable product characteristics, along with merchandising, makes them extremely appealing. Our intervention has therefore been designed to reduce awareness and attraction of discretionary products while expanding the awareness and attraction of substitute core products. ALPA is responsible for strategy implementation through its store managers.

The implementation intervention logic is based on the Behavior Change Wheel [34] and is expected to affect store manager behavior in relation to maintenance of the strategy through the following 5 functions: (1) Persuasion (ALPA management communicating support for and importance of the study to induce positive feelings of store managers to stimulate action), (2) Incentivization (ALPA management communicating benefits of the study to store managers and creating an expectation of reward in terms of health benefit to the community and recognition of ALPA as a leader in healthy food retailing), (3) Training (implementation setup team imparting skills to store managers on how to maintain the strategy), (4) Environmental Restructuring (ALPA management providing hands-on assistance to store managers to set up the strategy; ALPA management and store managers supporting the strategy), and (5) Enablement (ALPA management and research team showing support for the strategy and assisting store managers with troubleshooting of issues that may arise during implementation).

Ethical approval has been granted by the combined NT Department of Health and Menzies School of Health Research Human Research Ethics Committee (ref: HREC-2018-3048) and the Far North Queensland Human Research Ethics Committee (ref: HREC/18/QCH/23-1211).

### Eligibility and Recruitment

#### Recruitment of Stores

This research will be conducted in partnership with ALPA who, at the time of designing the study, managed 25 stores in 24 communities. The ALPA stores comprise 3 corporation types, as these are as follows: company owned stores (6 in Queensland), stores managed on behalf of Aboriginal store owners (12 in NT and 1 in Queensland), and member stores where community residents are the shareholders (6 in NT). Overall, 12 of these stores operate in communities where there is more than 1 store. All stores managed by ALPA will be eligible. Using stores managed by a single store association will help ensure uniform and high-level implementation fidelity. In the 1 community where there are 2 ALPA stores, both stores will be allocated together to intervention or control during randomization.

The recruitment process will commence with the study being first presented and discussed with the ALPA and the Island and Cape boards. These boards are able to give consent for the ALPA and Island and Cape owned stores. On approval from the ALPA board, contact will then be made with each of the stores in NT with a management agreement with ALPA and a meeting arranged with the respective store boards. A study story will be used to facilitate a face-to-face discussion with store board directors. This will be facilitated by ALPA personnel alone or together with a member or members of the research team. Local authority groups in each of the communities will be informed of the study via a letter describing the study purpose and indicating that the community store board will be invited to participate.

### Randomization

Consenting stores will be allocated to intervention or control using random number ranking, Stata version 15, StataCorp LLC.

Blinding. Blinding of store allocation is not possible for store managers, ALPA personnel responsible for intervention implementation, research staff administering the intervention, or customers and other community members. This will not impact the data collected for the primary and secondary outcomes, as the store sales data used to measure intervention effect are objective electronic data. In addition, objective measures in the form of photographs will be used to assess implementation fidelity.

### Intervention

Through our co-design approach with ALPA, the intervention (Table 1) builds on its new front-of-store and end-of-aisle strategy. It includes components that the ALPA nutritionist together with the ALPA merchandising and operations teams considered feasible and acceptable to trial from a business perspective and that the research team proposed to likely be the most effective.

A traffic light system based on the NT School Nutrition and Healthy Eating Guidelines [35] will be used to classify food types as healthy or less healthy, with discretionary food types flagged as red (less healthy).

### Implementation

The ALPA General Manager of Retail Services and ALPA Nutritionist, who are both study investigators, will use a store task list, and they will use a drinks fridge planogram (physical layout diagram) developed by ALPA with input of the study investigators on proportion of unhealthier to healthier drinks, to communicate to store managers and their area managers about what is required. The task list will be developed on the basis of merchandising practice observed and photographed for each intervention store at baseline, specifically tailored on the basis of each store’s unique premium high traffic areas such as counter, front- and end-of-aisles, and store entrance. The task lists will indicate the maximum number of shelf facings for sugar, sugar-sweetened beverages, sweet biscuits, and confectionery product categories, with a list of substitutable core food products to fill the space opened. Substitutable core products will be identified with ALPA and indicated to store managers in a 2-page reference guide. Fidelity to no price reductions on targeted products will be maintained from baseline, with no changes during the intervention period, via ALPA’s standard pricing procedures. ALPA will lead the implementation of the intervention and will support the training of its store staff in implementation procedures for each of the intervention stores at start-up. The store will be relayed (ie, implementation of strategy) in consultation with store managers by members of the research team with an ALPA area manager or the ALPA nutritionist with an ALPA area manager. Store managers will use photographs of the newly relayed store to communicate stocking procedures to store staff for strategy maintenance. Thereafter, store managers will be responsible for maintaining the intervention with the support of their area managers. We expect some local adaptation of the intervention because of heterogeneity in the physical design of each store and input on implementation from store directors, but ALPA will aim for standardization of the intervention delivery across stores. Strict monitoring of intervention compliance will be conducted by the research team (see the Implementation Evaluation section below). Any nonadherence identified through monitoring will be communicated immediately to the responsible store manager to correct.

**Table 1:** Intervention components.

Targeted discretionary items (all red table sugar, sugar-sweetened beverages, sweet biscuits, and confectionery; price, promotions, and place)	Healthier alternatives (all green and amber items; price, promotions, and place)
1. No promotional activity^a^ on discretionary products, including no price discounts, volume promotions (eg, 2 for 1 type offers), posters, shelf stripping, and fridge branding	—^b^
2. No misleading promotional activity (eg, fruit and vegetable fridge branding on a fridge containing confectionery, or no sugar shelf stripping on shelves with sugary drinks)	No misleading promotional activity (eg, fruit and vegetable fridge branding on a fridge containing confectionery, or no sugar shelf stripping on shelves with sugary drinks)
3. No visible availability at counter and high traffic areas^c^ of discretionary products (eg, front-and end-of-aisle displays)	Substitute visible availability of core products
4. Reduced facings^d^ (ie, number of identical products on a shelf)	Substitute facings of core products in the proximity of targeted product categories where the facings have been reduced
5. Reduced refrigerator space for targeted drinks^e^	Substitute refrigerator space for healthier drinks as follows: water, small units of unsweetened fruit juice, and artificially sweetened beverages
6. In stores with no non-Arnhem Land Progress Aboriginal Corporation competitor store in the community, no units more than 600 ml of targeted soft drinks permitted in refrigerators	—
7. Shelf stripping warning on target products and floor sticker indicating quantity of sugar in drinks	Floor sticker promoting water as the healthiest drink choice

^a^Price mark-downs with no signal of savings to the customer permitted on short-dated food and drink stock. Marked-down items not permitted in high traffic areas.

^b^Not applicable.

^c^Products considered at high risk of theft to remain at front of store, but in the least prominent location such as under the counter.

^d^Sweet biscuit facings reduced by half; table sugar facings reduced to 1 bay, no multipacks displayed, smaller units at eye level; and confectionery facings reduced by half and no increase in range permitted.

^e^Artificially sweetened drinks (diet drinks) were classified as amber, not red.

Control stores will be asked to continue usual store practice and told by ALPA that they will be supported to implement the Healthy Stores 2020 strategy at the study end if demonstrated to be effective.

### Governance

Decisions relating to study design and protocol, ethics requirements, and research dissemination have been and will continue to be made by the research investigator group who meets monthly. Overall, 3 working groups will be established, comprising research investigators and key stakeholders to oversee and advise on development, implementation, and evaluation of the (1) merchandising strategy, (2) customer intercept survey, and (3) qualitative study to identify the policy supports for scale-up of health-enabling policy action in stores. A communication and research dissemination strategy approved by the research investigator group will govern internal and external stakeholder communication. This includes feedback of study findings and lessons learned at the end of the study to the ALPA and Island and Cape boards and participating communities.

### Study Outcomes

#### Primary Outcome

The primary outcome is difference in free sugars (g/MJ) from baseline in intervention versus control stores derived from store sales data. Free sugars contributed 26% of total energy purchased from 20 community stores, more than double the World Health Organization recommendation of less than 10% of total energy intake [36]. Our outcome measures were informed by modeling the estimated impact of the intervention on both target product categories and nutrients, using 49 continuous weeks of nonintervention store sales data collected from 20 remote Indigenous communities who participated in the SHOP@RIC study.

#### Secondary Outcomes

We will assess the impact on store revenue as a secondary outcome. This measure was considered by ALPA as important in determining the impact on retail performance. A necessary outcome for ALPA is that revenue is maintained throughout the intervention. Retail measures useful in evaluating specific merchandising effects on business operations will be examined where data are available, including number of products purchased per transaction (basket size), number of unique transactions, and category share of store sales. We will also collect data on intervention costs, including costs associated with strategy material production, implementation, and evaluation.

Our use of store sales data captures all foods and drinks sold, enabling an assessment of total nutritional quality of purchases as well as exploring impact on specific product types. We will examine the impact on purchases of targeted discretionary products (table sugar, sugar-sweetened beverages, sweet biscuits, and confectionery), total discretionary products, and nontargeted products (eg, water, diet drinks, fruit, and vegetables). We will examine the impact on the nutritional content of all food and drinks sold, including total energy, energy density, and nutrient density.

### Implementation Evaluation

#### Merchandising Checklist

We will assess intervention implementation using a Merchandising Checklist and the Store Environment tool (Store Scout App). The Merchandising Checklist will capture the intervention activities. Store managers from control and intervention stores will be requested fortnightly during the 12-week intervention period to respond to a brief checklist of intervention components delivered by a member of the research team via phone, and they will be requested to provide photos of premium locations (hot spots for product displays, such as high traffic areas, defined for each store following baseline data collection) to verify degree of compliance with intervention components. Store managers will be asked to comment on observations about community-level incidents that they perceive may affect sales (eg, weather events or festivals). They will also be asked questions related to their perceptions of intervention implementation and effectiveness.

#### Store Environment Tool (Store Scout App)

Store Scout App (developed and piloted by our team in 2016 in 6 remote stores) assesses the overall store as a consumer environment (ie, the retail choice architecture), through measures of stocking and merchandising of 7 categories of food and drinks (including fruit and vegetables, drinks, snacks, meals and convenience food, breads and cereals, dairy products and eggs, and meat and seafood). We will train government and Aboriginal Community-Controlled Organization employed public health nutritionists to conduct assessments in control and intervention stores. The tool will be completed at the end of the baseline, intervention, and postintervention periods.

### Customer Intercept Survey Substudy

In the final 2 weeks of the intervention, we will conduct customer intercept surveys (1080 unique customers; 45 per store) from control and intervention stores to identify the following:

1. Differences among customers in discretionary food and drink purchasing in intervention and control communities.
2. Customer characteristics that predict discretionary product purchases.
3. Proportion of customers who regularly purchase food and drinks from other food outlets in or outside of the community.

Using a structured close-ended question survey in electronic format (iSurvey) with standardized scripts, trained surveyors with support from store staff will interview customers after they complete their purchase. Surveyors will be scheduled to survey customers at store front (postshop) over a period of 6 hours (3×2 hour sessions over the course of the day) per day for up to 3 days, to capture a broad spectrum of customers and shopping purpose, planned or unplanned, each subject to different levels of impulse shopping propensity. We consulted with ALPA to ensure this is feasible on the basis of number of transactions per day. We anticipate surveys to take 5 to 10 min per customer.

Upon exiting the store, customers will be invited to provide their receipt (a photograph of the receipt will be recorded) and respond to a short-item questionnaire to gather information on characteristics of the customer (age range, gender, shopping alone versus with child or others, and impulse shopping propensity). Data on payment method (eg, cash or card) and food shopping frequency at other retail outlets will also be collected.

### Data Collection

The data source for the primary and secondary outcome analysis will be weekly sales reports generated by ALPA for each store for the entirety of the study and sent electronically to the research team. These data will include product identifier (stock keeping unit or barcode), product description, quantity sold, and dollar value. Store products will be linked to nutrient data using a database that we have developed specifically for this purpose, which is mostly derived from the Australian Food, Supplement, and Nutrient Database [37], with discretionary food flagged from the Discretionary Food List developed by the Australian Bureau of Statistics [38]. We will use built-in, reliable data checking processes that we have used over the last decade in our research to assess and quantify the nutritional impact of sales.

### Analyses

#### Effect of Intervention

Longitudinal data analysis models will be used on fortnightly store sales data aggregated from weekly sales. This will enable the effect of the intervention to be expressed as a relative change. Analyzing fortnightly data reduces variation because of income cycles, as observed in our previous analyses with sales data [6]. All models will include random effects for the stores and fixed effects for fortnight and intervention. Within-store residuals will be assumed to have an autoregressive structure of order 1. We will report effect sizes (and 95% CI) together with the associated *P* values.

Descriptive statistics will be used to describe contextual data. To assess implementation fidelity (as described in the implementation evaluation), a dichotomous variable of high or low fidelity will be derived from the repeat measures collected throughout the study in intervention and control stores using the Merchandising Checklist. Contextual and qualitative data will be analyzed to determine factors influencing implementation.

A store environment global score will be derived from use of the Store Scout App and compared across the 3 time periods. We will assess the degree to which the intervention has impacted the consumer environment and been sustained at 24 weeks postintervention in control and intervention stores.

Analyses will be conducted according to the intention to treat principle applied at store level; sensitivity analyses will be conducted looking at the stores’ implementation fidelity. Statistical analyses will be performed using Stata version 15.

#### Customer-Level Response (Substudy)

Percent discretionary product to total food and beverage dollars (dependent variable) will be calculated for each receipt collected during the Customer Intercept survey. Associations between the dependent variable and dichotomous variables indicating exposure or no exposure to the intervention will be estimated using mixed-effects linear models. Effect of the intervention will be expressed in terms of percent difference relative to the control group adjusting for baseline differences in merchandising. Mixed models will include a random intercept for community to account for within community correlation (clustering effect). As this is a convenience sample of customers, the potential confounding effect of baseline exposure to merchandising of targeted products, age range, and gender will be explored by including these factors in the models. Store Scout will be conducted at baseline in control and intervention communities to provide a proxy of merchandising exposure at baseline. Multivariable linear mixed models (with random intercept for community) will be conducted to identify customer characteristics (eg, payment type) associated with purchase choice.

### Sample Size

#### Primary Outcome Measurement

Our intervention targets products that collectively account for 87% of free sugars from all product purchases in remote communities. The effects of the strategy are likely to be seen from the beginning of the intervention period, and we expect that sales (gram weight) of targeted products will be reduced by approximately 10% and free sugars (g/MJ) by approximately 8% to 9% throughout the intervention; we will test if this is sustained postintervention. A mean effect size of a 10% reduction in targeted discretionary product categories purchased is based on Batis et al (2016) [39], where an 8% tax applied by the Mexican government on nonessential energy-dense foods resulted in low socioeconomic status households purchasing on average 10.2% less taxed foods than expected (−44 [−72, −16] g per capita per month). Using 20 weeks of data for 20 remote stores (SHOP@RIC data), we found a 95% CI for the relative change of free sugars (g/MJ) for 2 randomly chosen groups of 10 stores to have width ±3.6% approximately. This anticipated precision is excellent for detecting an anticipated effect of the primary outcome of approximately 8% to 9%. A corresponding power calculation is not necessary [40]; however, the proposed study would likely have approximately 90% power to detect a 6% reduction in the primary outcome (free sugars g/MJ).

##### Customer Response

Under the assumption of an intracluster correlation equal to 0.01 and a cluster size equal to 45 (on the basis of 24 communities), we calculated a sample size allowing a study design of 1.4. With a sample of 1080 customers, and assuming a relative SD of 40% for the outcome discretionary product dollars of total dollars, the study will have 80% power to detect an 8% difference (from 50% in the control group to 42% in the intervention group) in the discretionary product dollars and total dollars at an alpha of .05.

## Results

As of August 2018, 20 stores consented to participate and were randomized to receive the intervention or continue usual practice. The 12-week strategy ended in December 2018. The 24-week postintervention follow-up will occur in May 2019. Results are expected for 2019.

## Discussion

### Principal Findings

There is strong evidence to show that merchandising is used to drive sales of discretionary foods [41]. The paucity of evidence on how to use merchandising techniques to reduce purchasing of discretionary products and nudge consumers toward healthier defaults is a large gap in our knowledge [42,43]. The limited available evidence suggests promise in reducing merchandising activity to restrict discretionary products and using merchandising activities to increase visibility and boost sales of targeted core foods [16]. Interest from retailers, including remote community store directors, to engage with researchers represents a unique and invaluable opportunity to address this evidence gap and cocreate knowledge. This trial will provide practical evidence needed to advance how public health can work with retailers to promote, implement, and evaluate health-enabling strategies in this private sector setting. As remote retailers’ decisions directly impact population diet, generating this evidence through collaboration with retailers and other important knowledge users will ensure its viability.

The outcomes will indicate the level of effectiveness and feasibility of the proposed strategy and identify reasons for these outcomes. We will then build on these results to make recommendations for policy, if appropriate, or for the next research study, to identify effective and feasible healthy retailing interventions. The evidence from this study will directly inform the nutrition policy of ALPA and will indirectly influence policies of other retail organizations and community stores through the leadership and influence of ALPA in remote retailing and through the broader effort of the Commonwealth Department of PM&C, which is responsible for food security in remote Indigenous Australia.

### Implications

Comprehensive multicomponent interventions are necessary to improve the quality of the customer food environment [44,45]. Our research will increase the health sector’s understanding of merchandising as a critical factor influencing behavior, one that with appropriate support, can be implemented by retailers. It will also help improve other food environment interventions led by practitioners and academics worldwide, particularly those in other remote populations who experience similar health disparities. Indigenous Australians, especially those who live in remote locations, experience substantially more preventable chronic diseases compared with other Australians. Improving diet is imperative for closing the Indigenous health gap, and improving dietary intake can benefit the future health of generations to come. A key contributor to the adverse diet quality in this population is the alarmingly high intake of the discretionary products that have proliferated the shelves in remote stores [28]. Food is predominantly sourced from community food retail stores, and many retail outlets operating in remote Australia are entirely community owned with store owners seeking in-store solutions to support healthier diets and combat diet-related diseases in a sustained way without negatively impacting on store sales. It is imperative that we work with retailers to provide high quality evidence to inform effective policy and practice for better health outcomes.

### Conclusions

Examining the impact of modifying retail food environments for improved diet and health outcomes is a rapidly expanding research area worldwide [41]. Globally, our team is one of only several research teams who is actively investigating the role of retail food environments in population health. The bringing together of decision makers and practitioners in health and retail sectors including ALPA personnel, with investigators to lead this research, who are involved in food retail environment research in urban, rural, and remote contexts in Australia and Canada, will help lessen the current issue of methodological heterogeneity in the measurement of food environment exposures and outcomes [12,46]. This will enable enhanced generalizability of accumulated evidence on the impact of retail food environments on diet into the future.

## Appendix

Multimedia Appendix 1 Peer review from funding agency.

Multimedia Appendix 2 Authors response to peer review.

## Abbreviations

ALPA: Arnhem Land Progress Aboriginal Corporation
MJ: megajoule
NHMRC: National Health and Medical Research Council
NT: northern territory
PM&C: Prime Minister and Cabinet
SHOP@RIC: stores healthy options project in remote indigenous communities
