# Supplementary material for: Reducing Retail Merchandising of Discretionary Food and Beverages in Remote Indigenous Community Stores: Protocol for a Randomized Controlled Trial
Source: JMIR Res Protoc. 2019 Mar 29;8(3):e12646. doi: 10.2196/12646 (PMC6538313; doi:10.2196/12646)
Supplement: Multimedia Appendix 1 [file resprot_v8i3e12646_app1.pdf]

Australian Government
National Health and Medical Research Council

NHMRC

GPO Box 1421 | Canberra ACT 2601
16 Marcus Clarke Street, Canberra City ACT 2600
T. 13 000 NHMRC (13 000 64672) or +61 2 6217 9000
F. +61 2 6217 9100
E. nhmrc@nhmrc.gov.au
ABN 88 601 010 284
www.nhmrc.gov.au

Associate Professor Julie Brimblecombe
julie.brimblecombe@menzies.edu.au

Dear Associate Professor Brimblecombe

Application ID: APP1138629
Type: Project
Application Title: Healthy Stores 2020: Reducing retail merchandising of discretionary food and beverages in remote Indigenous community stores

Thank you for applying for National Health and Medical Research Council (NHMRC) Project Grant funding commencing in 2018.

You are now invited to respond to assessor comments by submitting your Applicant Response (rebuttal). Assessor comments for your application are provided in the Assessor Report at Attachment A.

Your rebuttal must be uploaded into NHMRC's Research Grants Management System (RGMS) by **23:59 hrs AEST Friday, 14 July 2017** to ensure that it is provided to the relevant Grant Review Panel for consideration along with your application. Rebuttals received after this time will not be accepted. Instructions for submitting your rebuttal are provided in the *RGMS User Guide – Assessment Processes.*

Please ensure your rebuttal is a single PDF document that meets the size, length and formatting requirements outlined at Attachment B. Rebuttals that do not adhere to these formatting requirements will be excluded from consideration.

As your response is limited in length, please use your judgement in responding to assessor comments and address the most important issues. The rebuttal should be used to clarify queries raised by the assessors and not to propose changes to the project plan, methodology or team membership.

If you consider that your Assessor Report contains biased or inappropriate comments, you should contact your Research Administration Officer (RAO). RAOs may then contact projects@nhmrc.gov.au to raise your concerns and to seek NHMRC's advice on how they may be addressed.

It is important to raise potential concerns regarding the appropriateness of assessor comments during the Applicant Response (rebuttal) period. Complaints received after peer review will be difficult to address.

Applicants should continue to address other elements of the Assessor Report while NHMRC reviews concerns. RAOs will be notified of the outcome of NHMRC's review.

If you have questions regarding the rebuttal process, please contact NHMRC's Research Help Centre at help@nhmrc.gov.au or on 1800 500 983.

Yours sincerely

Research Grants
National Health and Medical Research Council

Encl.

Attachment A: Assessor Report
Attachment B: Applicant Response formatting requirements

GPO Box 1421 | Canberra ACT 2601
16 Marcus Clarke Street, Canberra City ACT 2600
T. 13 000 NHMRC (13 000 64672) or +61 2 6217 9000
F. +61 2 6217 9100
E. nhmrc@nhmrc.gov.au
ABN 88 601 010 284
www.nhmrc.gov.au

Attachment A: Project 2017 Assessor Report

**Application ID**	APP1138629
**CIA Name**	Associate Professor Julie Brimblecombe
**Application Title**	Healthy Stores 2020: Reducing retail merchandising of discretionary food and beverages in remote Indigenous community stores

<u>**Spokesperson**</u>
01. Scientific Quality
Consumer intercept survey completed by trained surveyors. Who are the trained surveyors?
Analysis includes copy of receipt. How many people take this? Sensitivities regarding Basic card in NT. Is this likely to make consumers wary of participation?

<u>**Spokesperson**</u>
02. Significance of the expected outcomes AND/OR Innovation of the concept
Poor social determinants combined with marketing techniques has led to poor consumer choices in many Indigenous remote communities. this is aggravating the increased risk of early onset complex chronic diseases. this research which is aiming for population level changes, beyond just focusing on individual choice, is likely to generate significant interest in many countries. the findings may well be of interest more broadly.

<u>**Spokesperson**</u>
03. Team quality and capability – relative to opportunity
Consists of established, mid career and an early career researchers with track records in undertaking population level interventions focused on improving food systems, as well as describing population obesity. Also includes Aboriginal CI with business and community engagement experience.

<u>**Spokesperson**</u>
Budget Comments
The PSP4 public health nutritionist is not described as having research skills. Wouldn't this be suitable as PSP3?

This is a three year study, with the largest costs in DRC due to travel to 25 remote communities. the costs appear well justified.

<u>**Spokesperson**</u>
Overall Comments
None provided.

<u>**Spokesperson**</u>
01. Scientific Quality
Hypothesis - H1a primary outcome is that intervention stores will have reduced sales of discretionary products (free sugars) compared to control stores and H1b secondary outcome no change from baseline in overall sales performance in both control and intervention stores.
The research aims are well stated and feasible to assess the impact of reducing discretionary product merchandising) by restricting visibility and/or availability) on consumer purchasing and retail performance in

remote Indigenous communities; and identifying the characteristics of store shoppers more likely to respond to the intervention, and analysing policy drives in remote NT and North Queensland to characterise the policy supports needed to scale up nutrition evidence uptake in retail stores in remote stores.
I think identifying the most appealing efficacious prevention/intervention to ensure ownership and a sense of choice is an important element that appears to missing in the aims even though it is mentioned later.The Methods and approach have been applied previously and tested. The primary and secondary aims are clear and align well with the outcomes that are being sought and measures. Overall the research proposal is well integrated and adequately developed. The main strengths of the proposal is the study design using a RCT designed with a 12 week baseline, a 12 week intervention and 24 week postvention across three different store types which are randomly selected and will enable to the team to account for store characteristics. The plan includes training local community people to undertake the store survey to address the second aim which is to determine consumer characteristics and to understand the knowledge, attitudes and behaviour change. A process evaluation built into the process to measure program fidelity, and a store environment tool to assess changes in store design.. In addition the qualitative methods to analyses the policy drivers to address the third aim.

Spokesperson
02. Significance of the expected outcomes AND/OR Innovation of the concept
Significance The project if successful has the potential to provide new knowledge about the importance of improving the store environment and purchasing policies to have a positive impact on the health outcomes. This project has the potential to show that address a range of chronic diseases as a which public health issues which requires a community wide and policy response rather that would help to prevent the need for high cost clinical interventions to address the growing illnesses such a kidney disease and diabetes with their attendant poor quality life outcomes.
The previous studies undertaken by the CIA and others have already shown there is high level of interest from other researchers, and community groups and policy makers in the research outcomes and resulted in journal publications. This interest is likely to increase and to result in highly influential publications and knowledge exchange given the up-scaling of this study across sites and the more extensive innovative and creative interventions which could change the whole diet and health and quality of life outcomes for Aboriginal communities across the Top End.
Importantly, it has the potential to influence current store purchasing and presentation practices approaches.

Spokesperson
03. Team quality and capability – relative to opportunity
The Track records of all CIs suggest they are capable of achieving the proposed study and has ability of having the appropriate mix of skills, expertise and experience of relevance to the research to deliver the project in terms and taking into account time commitment. Several Cis have national and international standing. Further CIE & CIG are emerging early career researchers with impressive outputs relative to opportunity.
Relevant research outputs and publications that illustrate innovation and significance and impact or outcomes of previous research achievements including on health care practice. The overall quality and impact of their publications are very relevant to the project.
The involvement of the ALPHA Chief Operating Officer and the Nutrition manager as AIs will enhance the feasibility and sustainability of the project. The team's research achievements to date confirm they have extensive experience to undertake a high level of health policy advocacy as well as an evident commitment to community and stakeholder engagement

Spokesperson
Budget Comments
The travel budget seems excessive and does not seem to allow for driving between communities but calculated for individual trips?

Spokesperson
Overall Comments
Overall the research proposal is well integrated and adequately developed. However, I think there needs to sensitivity to the history of the NT intervention. Perhaps replacing the word intervention with 'innovation' or 'strategy' to describe the research activities would avoid the risk of being perceived as imposing on communities 'for their own good'.
The study and its design tend the focus on the consumer characteristics rather than considering their perceptions of the value of the inventions including food education and the strategies to support people with sugar cravings and being hooked on products such as coke. What are the chances that people will end up travelling into closest towns etc – ie creating unintended consequences. Are these issues the team have considered?

External Assessor
01. Scientific Quality
The RCT design, simplicity and the narrow clear focus of the intervention allow the development of clear and clean objectives based on a simple hypothesis. This strength is further enhanced by defining one key nutrition

outcome to measure and the continuous data collection across the project - before during and after the intervention. However, some of the natural limitations of real world research influence the comparability of data from different stores and thus the number of potential variations in the nature and delivery of the intervention should be firmly controlled. Multiple elements in the intervention including those aimed at discouraging discretionary food purchases and those aimed at increasing core food purchases and including different strategies utilising positioning, price, and availability can lead to heterogeneous interventions in different stores. There is no opportunity to assess the potential effect of individual elements so every store should have the same intervention delivered in exactly the same way. In addition the different models of operation of stores may be another potential confounder requiring consideration.

Integrity of implementation checklist will collect information on observations about community level incidents and a measure of store owner/manager motivation and self-efficacy to initiate health promoting strategies but no there is no indication on how this information might influence the ongoing implementation or analysis of data. Similar issues apply to the consumer intercept study.

The consumer level response measures relative expenditure on discretionary food rather than free sugar intake which is the stated outcome measure. It is great to see the dissemination and policy and program implications integrated into the project. However, the policy analysis process is a little confusing and all depends on workshops , the nature and objectives of which are not clearly defined

External Assessor

02. Significance of the expected outcomes AND/OR Innovation of the concept

Improving nutrition within the broader Australian communities has enormous potential to contribute to reducing the growing burden of chronic disease and improve general health and wellbeing. Addressing diet and nutrition issues within indigenous communities is an extremely important objective critical to achieving improvements in the health gap between indigenous and non-indigenous Australians. Therefore any project that can help identify potential strategies to improve nutrition within indigenous communities warrants consideration and assessment. Despite the principles of instore merchandising being well used by marketers to influence consumer purchase patterns, such techniques have not been widely tested in terms of their capacity to impact positivity of consumer diet. This project is likely to be of high significance to policy makers and the community as there is a growing acceptance of the need to improve the food and nutrition environment if we are to improve the overall diet, nutrition and well-being of all Australians. The proposed intervention requires only minor modification to existing food retail systems and services but has the potential to positively impact on the diet and nutritional health of the whole community. The closed environment of remote communities facilitates implementation and allows a tighter research design but the findings may have implications for food retail in a wide range of environments including those controlled by government agencies.

Issues remain about how transferable and scalable the findings of research undertaken in remote stores will be to retail stores in larger urban environments under the control of more commercial interests.

External Assessor

03. Team quality and capability – relative to opportunity

This is a relatively young team of CIs composed of three young and emerging researchers together with 2 early to mid-career researchers guided by 2 more senior researchers and an experienced indigenous leader and community advocate. However, despite the relative youth of the research team they have demonstrated significant productivity to date relative to opportunity and have good track records in the under-explored research issues around nutrition interventions in a controlled retail environment.

More importantly the team have the experience and expertise necessary to undertake research on this issue in Aboriginal communities in remote locations. The teams possesses essential skills, understanding, existing relationships and demonstrated sensitivity that is more important than research experience in ensuring the success of this type of research.

There is potential to include additional investigators with complementary experience and expertise in retail marketing and in consumer behaviour change research.

External Assessor

Budget Comments

This is not a large budget request and each item is generally well described and justified in detail.

There is a modest request to cover costs of staff to co-ordinate and other staff costs are related directly to task associated with the collection, analysis and dissemination of data from the trial.

There is only minor costs for materials and consumables.

Travel is expensive. Although the proposed program of work is simple, the remote locations and the need to invest heavily in building community relationships and trust often involves considerable time and contact. This does add considerable expense in terms of travel. However some of the proposed travel cost could be reviewed or justified more clearly.

There is already agreement with ALPA to support and enable this study and the intervention is being undertaken by the stores themselves. Does this community involvement necessitate all the proposed visits from project staff; 1. prior to obtain consent, 2. immediately before to assess baseline conditions 3. During to conduct intercept interviews, 4. After to assess changes. Would it not be more efficient and build more capacity if community

members were trained to undertake some of these tasks?- especially as community store staff are being flown to Darwin to undertake training for the project.
Is it really necessary to fly international Cis to Darwin to provide training in conducting intercept surveys?

External Assessor
Overall Comments
This is an valuable and informative research project that deals with an important issue. The proposal is generally well considered and presented. Although the intervention will be assessed in real stores functioning in different environments and operating styles, there is potential to tighten aspects of the intervention to ensure a degree of homogeneity across stores.
The capacity building and knowledge exchange opportunities and process are highlighted but much of the work is still reliant on key data collection and implementation actions being undertaken by investigators travelling out to ach store. This may improve the data collection integrity but does impact on capacity building within communities and project costs associated with travel.
Closely following the implementation process and having an integrated process for engaging communities and policy makers into a process to determine the policy and practice implications are useful features of the proposed research.
Questions for investigators.
How will the data collected during the process evaluation be utilised? Will it provide immediate feedback to improve or tighten the implementation of the intervention or will it influence how the data is analysed or interpreted?
Does the team contain adequate experience and expertise in retail marketing and in consumer behaviour change research?

External Assessor
01. Scientific Quality
A well designed community level RCT. There is a strength with targeting the availability and visibility of discretionary food products.
Specific questions:
1. What proportion reduction of facings is expected of high sugar products vs alternatives?
2. Will control sites be offered the intervention in a subsequent period, if shown to be effective? This goes to feasibility with recruitment to the study, and need to provide 'something' in the control site.
3. It is noted that primary hypothesis is about reduction of sales of discretionary products high in free sugars. Will there be any way of determining or measuring if such products may be purchased elsewhere, away from the remote locations. Can the researchers comment on the likelihood of this, and whether there is a chance that members of the community could simply obtain these products from another location, even if the store sales of these products reduce in the local community.
4. Justification for sample size: The calculations are noted. I did wonder if there would be value in describing the data in terms of changes to absolute values, rather than limiting to changes to % energy contribution. If g of free sugars are reduced, then it is possible (and likely) that the total energy intake will be reduced (assuming no substitution with other energy containing foods), and thus the % energy from free sugars may actually be about the same, rather than reduced.
5. In the intercept surveys, will there be some questions about possible purchasing habits at other locations, other than the remote store location? (such as people visiting from elsewhere and bringing in different products)
6. With the intercept surveys and the calculations about % discretionary product to total food and beverage $, can the researchers comment on how they will account for multiple purchasing opportunities throughout the week. Will the snapshot of one receipt be sufficient to reflect % contribution to total?

External Assessor
02. Significance of the expected outcomes AND/OR Innovation of the concept
This study on food environment impacts has the potential to assist with building healthier food environments, and would need to be used as a range of techniques to improve health of population groups – especially needed in vulnerable population groups of Indigenous Australians in remote settings. There is a growing interest by policy makers about how we can use the food environments to support health, but generally speaking there has been little research in this area, so this would provide important evidence about the impact.

External Assessor
03. Team quality and capability – relative to opportunity
The team has a good mix of skills, track records and community members. It does include some early career researchers, but they provide important skills and/or links to communities and are required for good governance of the project and are therefore appropriate as investigators on the team.
CIA has extensive experience in conducting research with vulnerable population groups, and has demonstrated this with successful leading of other nationally competitive grants.
CIB is an international researcher with policy and food environment research expertise and adds important expertise to the project.

External Assessor

Budget Comments

The budget is large, but that is not unreasonable for a significant project working in remote locations of Australia. About half the budget on the salary and half on other costs. The salary requests for PSP4 commence at 2.4FTE and then decrease as the project proceeds. These requests seem reasonable.
The other half of costs are largely associated with travel expenses - this will be required in order to run the project.

External Assessor

Overall Comments

This project would provide important evidence about the impacts of food environment changes to remote community stores. The researchers have focused their attention on the reduction of discretionary food and beverage products, which provide a substantive contribution to energy and contribute to a large proportion of the household budget on food purchases - so this is an appropriate focus.

Questions on the project are documented in comments about scientific quality of the project, with the queries about:
1. What is planned for the 'control' stores.
2. Comments on likelihood of purchasing/ provision of food from elsewhere.
3. Free sugars, and impact on energy, with consideration to absolute free sugar and energy amounts (as opposed to % contribution).
4. Data from the intercept surveys and what can be obtained from a snapshot vs a more comprehensive investigation of purchasing habits. This may depend on the approach to analysis, such as whether sufficient to have mean data of the group vs distribution of purchasing habits. Can the researchers comment on this.

Indigenous criteria comments (if applicable)

Q01. Indigenous Criteria - Community Engagement

The team has well established record of engaging with community members, including Store Board members, Store managers, school and health centre staff. they have also demonstrated good community engagemnent in development of research proprosals in response to findings from previous studies.

Q02. Indigenous Criteria - Benefit

Improvement in the food retail environment is a critical component of improving health outcomes in remote Indigenous communities.

Q03. Indigenous Criteria – Sustainability and Transferability

the research team has proposed strategies for sustainability and transferability which appear feasible with likelihood of success. development of policy breifing papers to inform both retailers, nutritionists and government departments, as well as inclusion of a key government official from Prime Minister & Cabinet as an AI,

Q04. Indigenous Criteria – Building Capability

The team will work closely with ALPA Store managers and owners. they will provide training support for Indigenous store staff min merchandising healthy food options,, as well undertaking consumer surveys.

Q01. Indigenous Criteria - Community Engagement

There is evidence of a strong level of engagement with the relevant Aboriginal communities and Aboriginal health services to ensure the feasibility of effective Indigenous led implementation. Really need to have community-wide engagement to ensure this doesn’t come across to consumers that their choice is being comprised by the ‘balanda’ coming in and doing this to them. There is also evidence that the methods, objectives or key elements of the project have been formed, influenced or defined by Aboriginal people.The engagement of ALPA store boards and community leaders in informed consent for the activities and the RCT design over the period is a critical component. But is still subject to vagaries of community politics.

Q02. Indigenous Criteria - Benefit

The proposal shows evidence that Indigenous people living in remote communities have been exposed to an obesogenic environment that has impacted significantly on poor health outcomes – chronic diseases and poor nutrition. The CI team have shown in two previous (SHOP@RIC and GFD) that there is real potential to improve

diets and enhance health benefits in the short team with the likely indirect benefits in the long term direct. The benefits are clearly linked to NHMRC Road Map priorities.
Building of their previous work CI Team have demonstrates that Indigenous leaders in communities and store owners have been very engaged over time and have expressed concerns about the high level of sugar and poor food purchases and subsequent poor health outcomes. They have also expressed a real interest and need to create a healthy store environment to address these concerns. It is not as clear that community member have been involved determining the changes, however the results of the previous studies have confirmed that Aboriginal and Torres Strait Islander peoples in these communities are responsive to changing their food purchasing options when the opportunity is present.

Q03. Indigenous Criteria – Sustainability and Transferability
The proposal provides a convincing argument that the outcomes will have a positive, sustainable and transferrable and lasting impact on the health and wellbeing of Aboriginal and Torres Strait Islander peoples across diverse communities. Further there are four study design and dissemination mechanisms to ensure transferability, including policy and knowledge exchange workshops with Indigenous store owners and other key stakeholder groups, the findings of which will inform policy briefs and influence future store policies and government policy. The proposal includes a plan to support store owners to participate in knowledge translation and exchange based on information of positive stories collected over time to present at the 2019 Chronic Disease Network Conference. There are ample examples of activities that are designed to increase community understandings and involvement in the trial and to be part of the positive knowledge exchange process to promote community wide understanding and to attain the skills and capabilities to adopt healthy food practices. The links of Cis to the PM&C will help ensure the likely uptake of findings to ensure a more unified approach to promote a healthy store policy and services in remote communities.

Q04. Indigenous Criteria – Building Capability
The Proposal describes how Aboriginal and Torres Strait Islander people will benefit from developing the capability the store owners to implement more effective food security initiatives, and increasing the capacity of 12 local people to have regular employment by facilitating their training in Cert 11 retail skills. The knock- on benefits from this will occur through role modelling, and increased knowledge of, ownership and pride in establishing healthy food initiatives etc.
The involvement of CI Miles as an experienced Aboriginal researcher associated with the project will support researchers and stakeholders to understand local issues, and enhance understanding of the complexities and protocols at a local level and develop their capabilities to be more culturally responsive.

Attachment B: Applicant Response formatting requirements

**Component**	**Requirements**
Format	A single document converted into a PDF file that must not exceed 2Mb in size. Applicants and RAOs are advised to retain a copy of the Applicant Response (rebuttal), including a copy of the PDF file submitted to NHMRC.
File name	PDF file must be named in the following format: "Application ID - CIA Surname - Applicant Response.pdf" (for example: APP1098765 - Smith - Applicant Response.pdf).
Page Limit	No more than 2 pages. References and updates to the Chief Investigator's publication list must be included within the page limit.<br><br>Note: Applications that addressed the *Indigenous Research Excellence Criteria* (refer to https://www.nhmrc.gov.au/book/nhmrc-funding-rules-2017/6-assessment-criteria) will be permitted to use **an additional (third) page** to respond to the assessor comments provided for this component of their research proposal**.**
Paper Size	Standard A4 (210 x 297mm).
Header	The header is allowed outside the margin rules but must be at least 1cm from the top of the page.<br><br>The header must include the title "Applicant Response" in centred font with the Application ID in the top, right corner.
Margins	All margins must be at least 2cm.
Font	Times New Roman only. At least 12 point.
Line Spacing	Must be set to single or greater.
Character Spacing	Spacing must be set to normal. Scale must be set to 100%.
Web Links	Do not include links to additional information on websites.
Graphics	Graphics (pictures, diagrams etc.) may be included. Please note that the Applicant Response may be printed in black and white therefore colour graphics must be visible and labelled appropriately.
Tables	Tabulated information containing text is not considered to be an image or diagram. Text within tables must comply with the above requirements concerning fonts and spacing.
Labelling Graphs and Images	Axes of graphs and labels on images must be no less than 10 point, Times New Roman font. The description and/or legends of all graphs and images must comply with the above formatting requirements.
