# Supplementary material for: Reducing Retail Merchandising of Discretionary Food and Beverages in Remote Indigenous Community Stores: Protocol for a Randomized Controlled Trial
Source: JMIR Res Protoc. 2019 Mar 29;8(3):e12646. doi: 10.2196/12646 (PMC6538313; doi:10.2196/12646)
Supplement: Multimedia Appendix 2 [file resprot_v8i3e12646_app2.pdf]

The assessor's note the strength in design (SP2, EA1, EA2) and the relevance and importance of our trial in a setting where improving diet is a high priority with strong community engagement (all reviewers). Our team has achieved >12 published manuscripts since grant submission.

Clarity of consumer intercept survey methods (SP1) Pairs of trained surveyors (a store staff member, and CI or project nutritionist) will conduct surveys. ALPA personnel will have store cashiers (as per the SHOP@RIC trial) remind shoppers to take their receipt. Through engaging store staff in the study we are confident that shoppers will feel comfortable with participating. We naturally anticipate that some consumers will not feel comfortable to participate and will decline, and have accounted for this in our estimated participation rate of 32%-47%. (SP2) We will collect information on consumer's experience with the strategy (ie shopping experience satisfaction (p7).

Budget considerations (SP1) It is essential for this study that the project nutritionist has strong research skills, hence the request for a PSP4. (SP2, EA1)The travel budget was carefully considered to ensure appropriate community consultation, intervention and data integrity while maintaining reasonable project costs, and maximum time 'on the ground'. The flight budget was based on an average cost for all communities (NT drive $0; NT fly $800; QLD fly to Cairns/drive $400 and QLD fly $1750). To build local capacity and optimise community engagement and resource efficiency, store staff will be trained in implementation of the strategies and data collection.

Ensuring ownership of strategies? (SP2) The study was co-designed with ALPA. It builds on and will rigorously evaluate ALPA's 'point of sale and end of aisle policy' aimed at promoting healthier food purchases. As identified by SP2, the merchandising strategies were identified with ALPA to be the most appealing and efficacious. ALPA's knowledge and close local interaction (with ALPA board members being community members) was essential, and their contributions ensure ownership and sense of choice. Ownership of strategies will be further ensured with ALPA leading implementation of the strategy by training and coordinating store staff.

Terminology and sensitivity to NT intervention (SP2) We agree with SP2 and will be sensitive to this issue. We used the term within this application in our attempt to improve ease of understanding, as it is the most common descriptor for this study design in academia. In the communities, it will be referred to as the Healthy Stores Project.

Consideration of participants outshopping (SP2, EA2) Data on food shopping frequency at retail outlets outside of the community will be collected (see p7). The 24 communities are very remote typically located at least 100km from a nearby centre. Stores in the SHOP@RIC trial together provided 96% (55%-119%) of the population's energy requirements. We anticipate that outshopping will be minimal and have negligible impact at the population level.

Ensuring homogeneity of strategies across communities (EA1) We can confirm ALPA intends to standardise delivery across stores. Informed by evidence, including that of our recent SHOP@RIC trial and ALPA expertise, the intervention has been designed to assess a set of [combined] strategies to target the whole of merchandising. This approach will maximise dose and proof of concept, rather than assessing the effectiveness of each individual strategy *per se*. ALPA operations staff will lead implementation and communicate this to store managers and staff through planograms and an operational manual. There is some flexibility of individual strategies at the local level, (as we anticipated, see p6), to cater for individual store characteristics in this real-world setting. ALPA operations staff will support implementation through store visits and training of store staff in strategy implementation which will ensure an acceptable level of overall homogeneity.

Use of process evaluation data? (EA1) Data on context and implementation factors will aid interpretation of study findings and will inform the resourcing needs to effectively scale the research for ALPA (and others). Such data are essential to unpacking the acceptability of different strategy components. A particular dichotomous variable of high/low fidelity derived from the Merchandising Checklist data (see p7) will be used in mixed models of data from the consumer intercept survey to determine the effect of implementation fidelity on consumer level purchasing behaviour. The Store Environment Tool will provide a description of changes in retail architecture and contextualise results. Our experience is that process evaluation data is critical to both interpret research outcomes and assist in future intervention design.

Consumer level response vs. free sugar intake (EA1) Relative expenditure on discretionary food will assess consumer level response to the intervention. This will assess the impact of the intervention on discretionary food/drinks. Our primary outcome measure for the population response focuses on free sugar intake where objective sales data will also be used.

Clarity of policy analysis process (EA1) The policy analysis process will initially identify key points where remote food supply decision-making of key stakeholders (eg retailers, nutritionists, other government officers) is influenced. Opportunities for uptake of nutrition evidence in programs and policy will then be identified. Such qualitative assessment combined with other evidence on strategies to promote healthy food purchasing will inform a series of policy briefing papers that will be presented at a policy options workshop and finalised at the knowledge exchange at the end of project event. It will offer a framework to scale-up sustainable nutrition policy for remote stores.

Transferability of findings (EA1) Through engagement with ALPA and the Commonwealth Department of Prime Minister and Cabinet, our research has potential to impact on store policy across 175 stores in remote Australia. Improving the health of remote Indigenous Australians is a recognised standalone Government priority. Our findings will also have direct translation to similar contexts overseas and will advance (local and global) knowledge on retail food environments.

Future support for control stores (EA2) During recruitment, community leaders will be fully informed of randomisation and that ALPA will support control stores to implement initiatives to support healthy eating post-trial (see p5). Representatives of control communities will have opportunities to share in the knowledge exchange event and to discuss healthy eating initiatives through face-to-face meetings with each community at the end of the project.

Complementary expertise in retail marketing and consumer behaviour change research (EA1) AI Greenacre is a Lecturer in Marketing at Monash University. His research focuses on issues of consumer perceptions and decisions within the broader Consumer Behaviour track. He regularly works with retailers, giving him a working knowledge of retail practices. CIs Mah and Minaker, both knowledge leaders in consumer behaviour research, will provide training in consumer intercept survey methodology and participate in the policy drivers analysis workshop during their visit, thus justifying the cost of overseas travel (EA1).

Proportion reduction of facings in high sugar products vs alternatives? (EA2) Our aim is for facings of sugar *per se*, sweet biscuits and confectionery to be reduced by 50%. This was identified by ALPA as feasible and substantial in influencing consumer purchasing.

Could absolute rather than relative (to energy) reporting of free sugars be used? (EA2) Although we predict that shoppers will reduce absolute purchases of high sugar discretionary products, we anticipate some level of energy substitution. We reported with our SHOP@RIC trial that total dollar spend did not change with the price discount. Therefore, energy change will not necessarily correspond with reductions in high sugar discretionary products, which would limit the interpretation of an absolute measure. A relative measure of change will allow us to more definitively describe intervention impact with regard to overall energy intake. Furthermore a relative measure allows for a comparison of effect with the WHO target of <10% of free sugars from energy. Absolute values are included as secondary outcome measures and will be used to assess if there has been masking of the effect due to a potential simultaneous reduction in both free sugars and energy.

What can be obtained from a snapshot vs more comprehensive investigation of purchasing habits? (EA2) The consumer intercept method is designed as a cost-effective method for capturing the characteristics of shoppers that predict discretionary product purchases and/or are associated with a greater response to the intervention. The snapshot of discretionary food/ drinks purchasing will be taken across all communities, on multiple days (n=3) and for multiple time periods (3 x 2 hr sessions) to capture the breadth of shopper types and a range of shopping purposes (planned or unplanned) (see p7). To minimise repeat surveys, shoppers who respond yes to having already participated in the survey will be excluded. This novel snapshot data on secondary measures will address a critical knowledge gap on consumer purchasing behaviour in remote Indigenous communities and inform future studies.

Indigenous criteria

We thank the assessors for their comments noting our teams well established record of engaging with community members (ATSI 1); that our proposal demonstrates a strong level of engagement with the relevant Aboriginal communities and Aboriginal health services to ensure the feasibility of effective Indigenous led implementation (ATSI 2); and, that it is evident that the methods, objectives or key elements of the project have been informed, influenced or defined by Aboriginal people (ATSI 2). We agree that the role of CI Miles, an experienced Aboriginal researcher with remote retail business and community engagement expertise is important to supporting our team and stakeholders to understand local issues, enhancing understanding of the complexities and protocols at a local level, and developing the team's and stakeholders capabilities to be more culturally responsive (ATSI 2).

Ensuring community engagement (ATSI 2) We are confident that our long-term and strong relationship with ALPA and the engagement activities we have planned at study start-up, including the engagement of the ALPA store board and community leaders in informed consent and the training and involvement of store managers and staff in strategy implementation and data collection, will facilitate community-wide support for the strategies. Our ongoing interaction with ALPA will ensure the project resilience needed to respond in an effective and appropriate way to the challenges that may present at the community level.

Points raised and addressed:

Sensitivity to NT intervention
(SP2) Perhaps replacing the word intervention with innovation or strategy to describe the research activities would avoid the risk of being perceived as imposing on communities for 'their own good'

Ownership of strategies
(SP2) I think identifying the most appealing efficacious prevention/ intervention to ensure ownership and a sense of choice is an important element that appears to be missing in the aims even though it is mentioned later.

Control stores
(EA2) Will control sites be offered the intervention in a subsequent period, if shown to be effective? This goes to feasibility with recruitment to the study, and need to provide 'something' in the control sites

Homogeneity of strategies across communities
(SP2) Some of the natural limitations of real world research influence the comparability of data from different stores and thus the number of potential variations in the nature and deliver of the intervention should be firmly controlled. Multiple elements in the intervention including those aimed at discouraging utilising position, price, and availability can lead to heterogeneous intervention in different stores. There is no opportunity to assess the potential effect of individual elements so every store should have the same intervention delivered in exactly the same way. In addition the different models of stores may be another potential confounder requiring consideration.

(EA1) Although the intervention will be assessed in real stores functioning in different environments and operating styles, there is potential to tighten aspects of the intervention to ensure a degree of homogeneity across stores.

Use of process evaluation data
(EA1) Integrity of implementation checklist will collect information on observations about community level incidents and a measure of store owner/ manager motivation and self efficacy to initiate health promotion strategies but no indication of how this information might influence the ongoing implementation or analysis of data. Similar issues apply to the consumer intercept survey

(EA1) How will the data collected during the process evaluation be utilised? Will it provide immediate feedback to improve or tighten the implementation of the intervention or will it influence how the data is analysed or interpreted?

(EA2) What proportion reduction of facings is expected of high sugar products vs alternatives Outcome measures

Outcome measures
(EA1) The consumer level response measures relative expenditure on discretionary food rather than free sugar intake which is the stated outcome measure.

Free sugars and impact on energy
(EA2) Justification for sample size: The calculations are noted. I did wonder if there would be value in describing the data in terms of changes to absolute values, rather than limiting to changes to % energy contribution. If g of free sugars are reduced, then it is possible (and likely) that the total energy intake will be reduced (assuming no substitution with other energy containing foods), and thus the %energy from free sugars may actually be about the same, rather than reduced.

Consumer intercept survey
(SP1)Who are trained surveyors
(SP1) How many people take their receipt
(SP1) Sensitivities regarding basic card – will this make people wary of participation
(EA2) How data might influence ongoing implementation or analysis of data
(EA2) With the intercept surveys and calculations about % discretionary product to total food and beverage $, can the researchers comment on how they will account for multiple purchasing opportunities throughout the week. Will the snapshot of one receipt be sufficient to reflect % contribution to total?

(SP2) The study and its design tend to focus on the consumer characteristics rather than considering their perceptions of the value of the inventions including food education and the strategies to support people with sugar cravings and being hooked on products such as coke.

Snapshot vs more comprehensive investigation of purchasing habits
(EA2) Data form the intercept surveys and what can be obtained from a snapshot vs more comprehensive investigation of purchasing habits. This may depend on the approach to analysis, such as whether sufficient to have mean data of the group vs distribution of purchasing habits. Can the researchers comment on this

Outshopping
(SP2) What are the chances that people will end up travelling into closest towns – ie creating unintended consequences. Are these issues the team have considered?

(EA2) It is noted that primary hypothesis is about reduction of sales of discretionary products high in free sugars. Will there be any way of determining or measuring if such products may be purchased elsewhere, away from remote locations. Can the researchers comment on the likelihood of this, and whether there is a chance that members of the community could simply obtain these products from another location, even if the store sales of these products reduce in the local community.

(EA2) In the intercept surveys, will there be some questions about possible purchasing habits at other locations other than the remote store location? (such as people visiting from elsewhere and bringing in different products).

Policy analysis
(EA1) The policy analysis process is a little confusing and all depends on workshop, nature and objectives of which are not clearly defined

Transferability
(EA2) Issues remain about how transferable and scalable the findings of research undertaken in remote stores will be to retail stores in larger urban environments under the control of more commercial interests

Budget
(SP1) The PSP4 public health nutritionist is not described as having research skills. Wouldn't this be suitable as PSP3?

(SP2) The travel budget seems excessive and does not seem to allow for driving between communities but calculated for individual trips

(EA1) Travel is expensive. Although the proposed program of work is simple, the remote locations and the need to invest heavily in building community relationships and trust often involves considerable time and contact. This does add considerable expense in terms of travel. However some of the proposed travel cost could be reviewed or justified more clearly. There is already agreement with ALPA to support and enable this study and the intervention is being undertaken by the stores themselves. Does this community involvement necessitate all the proposed visits from project staff 1. Prior to obtain consent 2. Immediately before to assess baseline conditions 3 During to conduct intercept interviews; 4. After to assess changes. Would it not be more efficient and build more capacity if community members were trained to undertake some of these tasks? Especially as community store staff are being flown to Darwin to undertake training for the project. Is it really necessary to fly international CIs to Darwin to provide training in conducting intercept surveys? The capacity building and knowledge exchange opportunities and process are highlighted but much of the work is still reliant on key data collection and implementation actions being undertaken by investigators travelling out to each store. This may improve the data collection integrity but does impact on capacity building within communities and project costs associated with travel.

Complementary expertise in retail marketing and consumer behaviour change research
There is potential to include additional investigators with complementary experience and expertise in retail marketing and in consumer behaviour change research

Aboriginal and Torres Strait Islander Criteria
Community engagement
(ATSI 1) There is evidence of a strong level of engagement with the relevant Aboriginal communities and Aboriginal health services to ensure the feasibility of effective indigenous led implementation. Really need to have community-wide engagement to ensure this doesn’t come across to consumers that their choice is being compromised by ‘balanda’ coming in and doing this to them.

There is evidence that the methods, objectives or key elements of the project have been formed, influenced or defined by Aboriginal people. The engagement of ALPA store boards and community leaders in informed consent for the activities and the RCT design over the period is a critical component. But is still subject to vagaries of communities politics

It is not as clear that community members have been involved in determining the changes, however the results of the previous studies have confirmed that Aboriginal and Torres Strait Islander peoples in these communities are responsive to changing their food purchasing options when the opportunity is present.

Positive remarks:

Scientific quality
Research aims well stated and feasible (SP2)
Methods and approach applied previously and tested (SP2)
Aims are clear and align well with the outcomes (SP2)
Research proposal is well integrated and adequately developed (SP2).
Main strengths is the study design using a RCT (SP2)
The RCT design, simplicity and narrow clear focus of the intervention allow the development of clearn and clean objectives based on a simple hypothesis (EA1)
Proposal is generally well considered and presented (EA1).
A well designed community level RCT (EA2).
Appropriate focus on discretionary foods (EA2).

Strong level of engagement to ensure the feasibility of effective Indigenous led implementation (IC2).
Evidence that the methods, objectives or key elements of the project have been informed, influenced or defined by Aboriginal people (IC2).

Track record
Consists of established mid and early career researchers with track records in undertaking population level interventions – Eddie's experience (SP1)
Track records – capable of achieving the proposal study and appropriate mix of skills, expertise and experience – emerging ECFs with impressive outputs relative to opportunity (SP2)
Involvement of ALPA COO and nutrition manager will enhance feasibility and sustainability of the project (SP2)
Teams research experience confirm they have extensive experience to undertake a high level of health policy advocacy as well as an evident commitment to community and stakeholder engagement (SP2).
Team have demonstrated significant productivity to date and have good track records in the under explored research issues (EA1).
Necessary experience and expertise to undertake research on this issue in Aboriginal communities (EA1).
The team possesses essential skills, understanding, existing relationships and demonstrated sensitivity that is more important than research experience in ensuring success of this type of research (EA1).
Team has a good mix of skills, track records and community emmbers (EA2).
EC researchers provide important skills and are appropriate as investigators on the team (EA2).
Well established record of engaging with community members (IC1).
Demonstrated good community engagement (IC1).
Involvement of CI Miles(IC2).

Budget
Costs appear well justified (SP1)
Not a large budget and each item is well described and justified in detail (EA1).
Budget is not unreasonable; request seem reasonable (EA2).

Significance/innovation
Likely to generate significant interest in many countries and findings be of interest more broadly (SP1)
Potential to provide new knowledge about the importance of addressing issues that require a community wide and policy response (SP2)

Highly influential publications and knowledge exchange that could change the whole diet and ehalth etc (SP2).
Improving nutrition within the broader Australian communities has enormous potential to contribute to reducing the growing burden of chonic disease and improve general health and wellbeing (EA1).
Likely to be of high significance to policy makers and the community as growing acceptance of the need to improve the food and nutrition environment (EA1).
Proposed interventions require only minor modification to existing food retail systems and services but has the potential to positively impact on the diet and nutritional health of the whole community (EA1).
Findings may have implications for food retail in a wide range of environments including those controlled by government agencies (EA1).
Valuable and informative research project that deals with an important issue (EA1).
This project would provide important evidence about the impacts of food environment changes to remote community stores (EA2)
There has been little research in this area of how to use food environments to improve health – so this would provide important evidence about the impact (EA2).
Improvement in food retail environment is a critical component of improving health outcomes in remote Indigenous communities (IC1).
Benefits are clearly linked to NHMRC road map priorities (IC2).
Real potential to improve diets and enhance health benefits (IC2).
Convincing argument that the outcomes will have a positive, sustainable and transferrable and lasting impact on the health and wellbeing of ATSI across diverse communities (IC2).

Translation

Having an integrated process for engaging communities and policy makers into a process to determine the policy and practice implications are useful features (EA1).
Potential to assist with building healthier food environment and could be used as a range of techniques to improve health of population groups (EA2).
Research team has proposed strategies for sustainability and transferability which appear feasible with likelihood of success (IC1).
Development of policy briefing papers as well as key government official (IC1).
Dissemination mechanisms to ensure transferability (IC2)
Proposal provides support to support store owners to participate in knowledge translation and exchange (IC2).
Ample examples of activities that are designed to increase community understanding and involvement in the trial ….to attain the skills and capabilities to adopt healthy food practices (IC2).
Links of CIs to PM&C will help ensure the likely uptake of findings to ensure a more unified approach to promote a healthy store policy and services in remote communities (IC2).
Knock on benefits of training local store staff (IC2).

Maximum 1 page (only to be used for comments on Indigenous Criteria)

Indigenous Criteria
